# Admission platelet count and indices as predictors of outcome in children with severe Sepsis: a prospective hospital-based study

**DOI:** 10.1186/s12887-020-02278-4

**Published:** 2020-08-19

**Authors:** Samira Z. Sayed, Mohamed M. Mahmoud, Hend M. Moness, Suzan O. Mousa

**Affiliations:** 1 Pediatric Department, Children’s University hospital, Faculty of Medicine, Minia University, El-Minya, Egypt; 2 Clinical Pathology Department, Children’s University hospital, Faculty of Medicine, Minia University, El-Minya, Egypt

**Keywords:** Severe sepsis, Platelet indices, Thrombocytopenia, Mean platelet volume, Platletcrit, platelet ratios

## Abstract

**Background:**

Sepsis is still one of the main causes of infants and children mortality especially in developing, economically challenged countries with limited resources. Our objective in this study was to determine, the prognostic value of platelet count, mean platelet volume (MPV), platelet distribution width (PDW) and plateletcrit (PCT) in critically ill infants and children with severe sepsis, as they are readily available biomarkers, that can guide clinicians during managing of severe sepsis.

**Methods:**

Sixty children were included; they were diagnosed with severe sepsis according to the international pediatric sepsis consensus conference criteria. At admission to Pediatric intensive care unit, complete blood count with platelet count and parameters (MPV, PDW and PCT) and C-reactive protein (CRP) level were determined for all children. Also, assessment of the Pediatric Risk of Mortality (PRISM III) score was done to all. These children were followed up till discharge from hospital or death. Accordingly, they were grouped into: (1) **Survivor group:** included 41 children. (2) **Non-survivor group:** included 19 children.

**Results:**

Platelet count and PCT were significantly lower (*p* < 0.001) and MPV was significantly higher in non-survivor than survivors (*p* = 0.004). MPV/PLT, MPV/PCT, PDW/PLT, PDW/PCT ratios were found to be significantly higher in the non-survivors than survivor (*p* < 0.001 in all). PCT with sensitivity = 94.74%, was the most sensitive platelet parameter for prediction of death, while MPV/PCT was the most sensitive ratio (sensitivity = 94.7%).

**Conclusion:**

Thrombocytopenia, platelet indices and their ratios, especially plateletcrit and MPV/PCT, are readily available, sensitive, prognostic markers, that can identify the severe sepsis patients with poorest outcome.

## Background

Sepsis is a major childhood disease both in terms of frequency and severity, and severe sepsis is still considered the main cause of death from infection in childhood. The prevalence of severe sepsis and septic shock among hospitalized children ranges from 1 to 26%. Mortality is high, ranging from 5% in developed countries reaching up to 35% in developing countries [1].

Although sepsis is considered a worldwide public health problem, it is still not tracked in the Global Burden of Disease report published by the WHO and World Bank [2], which is one of the most important sources of information for health policies decision-making in the world [3].

The absence of a well-established sepsis definition is a major obstacle to sepsis epidemiology in children. So, the third sepsis consensus conference (Sepsis-3) in 2016 published updated definitions for sepsis and septic shock that reflect the evolving understanding of sepsis pathobiology. Sepsis was defined as a ‘dysregulated host-response’ to infection leading to ‘life-threatening organ dysfunction’. Importantly, the foundation for this definition was no longer inflammation alone but a lack of immune homeostasis [4].

Unfortunately, definitions frequently provide limited value clinically; thus, ‘Sepsis-3’ recommends new clinical criteria for the rapid recognition of infected patients likely to suffer poor outcomes (ICU admission, prolonged length of stay, increased mortality) characteristic of sepsis, rather than uncomplicated infections [5]. Many studies performed in both developing and developed countries have shown that mortality from sepsis is high and is associated with delayed diagnosis, late treatment, and nonadherence to the treatment guidelines. Reducing mortality from pediatric sepsis is a worldwide challenge [1].

In the meantime, there are accumulating evidence about the important role of platelets in the inflammatory process, microbial host defense, wound healing, angiogenesis, and remodeling in addition to their contribution to hemostasis and thrombosis [6]. Some proteins released from platelet granules influence vascular wall and immune cell function [6–9]**,** other proteins are microbicidal and antibacterial [8]. Other studies demonstrated the important role platelet play in synthesis and release of vascular endothelial growth factors that is involved in tumor angiogenesis in addition to inflammation in tumor pathogenesis [10].

In recent years, the number of studies suggesting that the platelet and their indices can be used as inflammatory markers in cancer cases in addition to cardiovascular, cerebrovascular, inflammatory and thromboembolic diseases is increasing by the time [11].

In this study, our objective was to determine the prognostic value of thrombocytopenia and platelet indices (MPV, PDW and PCT), in critically ill infants and children with severe sepsis. As they are readily available biomarkers, most clinicians can make good use of, especially in developing countries where most cases of sepsis are.

## Subjects and methods

### Subjects

This cross-sectional hospital-based study was conducted on infants and children, who were admitted to PICU of Minia University Children and Maternity Hospital. The study was conducted during the period from July 2018 till January 2019.

We included in this study all patients who were diagnosed with severe sepsis according to the international pediatric sepsis consensus conference, which included the following criteria: - Sepsis plus one of the following: cardiovascular organ dysfunction or acute respiratory distress syndrome or two or more other organ dysfunctions [12]***.*** We excluded from our study patients admitted to pediatric intensive care unit (PICU) with causes other than severe sepsis e.g. intracranial hemorrhage, encephalopathy, hematological malignancy or DIC directly related to a malignant disorder. We also excluded patients whom parents refused to participate in the study.

### Sample size calculation

Sample was calculated to be 51 by sample size calculation GPower program at power of 80% and significance level less than 0.05. In a similar previous study by Golwala et al. 2016 [13], they calculated a sample size of 34 (17 non-survivors and 17 survivors) for a study power of 80% at the 5% level of confidence. Their prediction was based on results of a canine model [14].

All studied patients’ data was collected prospectively. Clinical characteristics and laboratory data collected during the first 24 h of hospital admission were used to determine The Pediatric Risk of Mortality III score (PRISM III score) [15]. Data was collected by the attending PICU resident and verified by one of our study team. PRISM score is used for prediction of mortality in pediatric patients. Recently, the physiologic status as measured with PRISM variables and their ranges could be used to simultaneously estimate morbidity and mortality risk [16]. The included subjects were followed up till discharge from hospital or death. Accordingly, they were grouped into: (1) **Survivor group:** children in this group were discharged from hospital after surviving severe sepsis attack. (2) **Non-survivor group:** this group included patients who expired during the course of severe sepsis.

## Methods

Blood samples were drawn from the patients upon admission to the PICU under complete aseptic condition for the following workup: arterial blood gases, random blood glucose, complete blood count, C-reactive protein (CRP), serum electrolytes, liver function tests, kidney function tests, prothrombin time, partial thromboplastin time, and blood culture. Cerebro-spinal fluid (CSF) culture and urine culture were only done when clinically indicated complete blood count (CBC) including platelet count and indices was done by fully automated cell counter Sysmex KX-21 N {TAO Medical Incorporation, Japan). CRP is widely used as a traditional prognostic marker for sepsis. It was assayed by NycoCard Reader II (Axis-Shield PoC AS, Oslo, Norway). CRP levels < 6 mg/dl were considered normal. Routine chemistry tests (blood glucose, renal function and serum electrolytes) were performed using fully automated chemistry analyzer Konelab 60i (Thermo Scientific, Finland). Prothrombin time (PT) and activated partial thromboplastin time (aPTT) were determined by using Fully automated coagulometer **STAGO** (Diagnostic STAGO – France). Blood samples for ABG were collected in heparin tubes and were determined by ABL90 FLEX blood gas analyzer (Radiometer Medical Apps, Denmark).

ABG, renal function (serum creatinine and blood urea nitrogen (BUN)), PT, aPTT, serum potassium and serum glucose level were used in calculation of PRISM III score.

PRISM score and CRP were determined for all patients to compare platelet count and indices with them.

Any additional laboratory or radiological imaging (chest x-ray, brain computed tomography (CT)) were ordered on case-by-case basis.

### Statistical analysis

The collected data were coded, tabulated, and statistically analyzed using SPSS (Statistical Package for Social Sciences) software version 25. Descriptive statistics were done for parametric quantitative data by mean ± standard deviation, and for non-parametric quantitative data by median and interquartile range (IQR), while they were done for categorical data by frequency and percentage. Normality of distribution of the data was tested by Kolmogorov Smirnov test. Analyses were done for non-parametric quantitative data using *Mann Whitney test* between the two groups. Correlations between PRISM score with CRP and other platelets parameters were done using *Pearson’s correlation coefficient*.

*ROC (Receiver Operating characteristic) curve* analysis of PRISM score, CRP and platelet parameters were done to determine Area Under Curve, optimal cutoff point, sensitivity, specificity, positive predictive value (PPV), negative predictive value (NPV) and accuracy for prediction of death in patient with sepsis. Using the cutoff values from ROC curves analyses, the odds ratio with 95% confidence interval was calculated using logistic regression analyses. The level of significance was taken at *p < 0.05.*

## Results

**Fig. 1:**
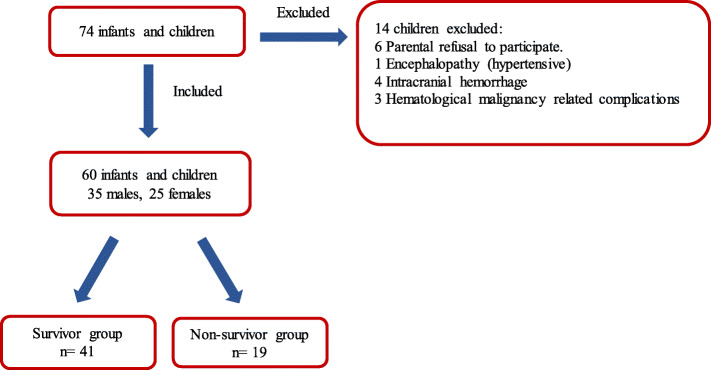
Flowchart of the enrollment of severe sepsis patients

During the study period, seventy-four infants and children were initially diagnosed as having severe sepsis upon admission to PICU, but only 60 patients were included in this study. As, fourteen patients were excluded: parents of six children refused to participate in the study and eight patients were excluded by the exclusion criteria (Fig. 1).

The sixty children included were 35 (58.3%) males and 25 (41.7%) females, with a mean age of 11.6 ± 7.5 months. Table 1 shows clinical, radiological, PRISM score and blood culture results of the included patients.

**Table 1:** Clinical, radiological, PRISM score and blood culture organism of the studied patients

		***n*** = 60
**HR** (beat /min)	*Mean ± SD*	162.5 ± 16.3
**RR** (cycle/min)	*Mean ± SD*	54 ± 15.9
**BP** (mm Hg)	*Normal* n (%)	11(18.3%)
	*Hypotension* n (%)	49 (81.7%)
**Hepatomegaly**	*No* n (%)	15 (25%)
	*Yes* n (%)	45 (75%)
**Chest finding**	*No* n (%)	22 (36.7%)
	*Yes* n (%)	38 (63.3%)
**CXR**	*Normal* n (%)	22 (36.7%)
	*Pneumonia* n (%)	37 (61.7%)
	*Empyema* n (%)	1 (1.6%)
**GCS**	*Mean ± SD*	8.9 ± 2.3
**Ventilatory support**	*None* n (%)	11 (18.3%)
	*Non-invasive* n (%)	22 (36.7%)
	*Invasive* n (%)	27 (45%)
**(+ve) inotropic support**	*No* n (%)	33 (55%)
	*Yes* n (%)	27 (45%)
**PRISM score**	*Median / (IQR)*	17/ (10–29)
**Blood culture organism**	*Staphylococcus aureus* n (%)	8 (13.33%)
	*Klebsiella* n (%)	13 (21.67%)
	*Streptococcus pneumoniae* n (%)	15 (25%)
	*Escherichia coli* n (%)	10 (16.67%)
	*Pseudomonas aeruginosa* n (%)	4 (6.67%)
	*Entrobacter* n (%)	7 (11.67%)
	*No growth* n (%)	3 (5%)

*HR* heart rate, *RR* respiratory rate, *BP* blood pressure, *CXR* chest x-ray, *GCS* Glascow coma scale, *PRISM* Pediatric risk of mortality score

We followed-up the patients till they were discharged from the hospital or died by complications of severe sepsis. Forty-one patients were discharged from the PICU. They were 24 (58.5%) males and 17 (41.5%) females with a median/ (IQR) age of 11/ (4–15) months. While 19 patients did not survive and died from complications of severe sepsis. They were 11 (57.9%) males and 8 (42.1%) females with a median/ (IQR) age of 12/ (2–15) months. Age and sex differences between the two studied groups showed statistical insignificance, as *p* > 0.05.

We compared the PRISM score, CRP, platelet count and platelet indices of the survivor versus the non-survivor. The PRISM score and CRP level were significantly higher in non-survivor children than those who survived, as *p* < 0.001, with odds ratio of 2.1 (95% CI: 1.1–2.9) for PRISM score and 1.01 (95% CI: 1.008–1.03) for CRP. While, platelet count and plateletcrit (PCT) were significantly lower in non-survivor than survivors, as *p <* 0.001 in both. Their odds ratios were 0.989 (95% CI: 0.982–0.996) and 0.1 (95% CI: 0.1–0.15) respectively. Meanwhile, the non-survivors had significantly larger mean platelet volume (MPV) than the survivors, as *p* = 0.004 (Table 2). MPV odds ratio was 1.9 (95% CI: 1.005–2.4).

**Table 2:** PRISM score, CRP, platelet count and parameters of the children with severe sepsis according to their outcome

Variable	Survivors	Non-survivors	p	Odds ratio (95% CI)
	***n*** = 41	***n*** = 19		
**PRISM** *Median / (IQR)*	11/ (10–15.5)	26/ (24–40)	< 0.001*	**2.1 (1.1–2.9)**
**CRP** (mg/dl) *Median / (IQR)*	96/ (48–96)	192/ (96–192)	< 0.001*	**1.01 (1.008–1.03)**
**PLT** (10^3^/ml) *Median / (IQR)*	265/ (115.5–381.5)	73/ (43–104)	< 0.001*	**0.989 (0.982–0.996)**
**MPV (fl)** *Median / (IQR)*	7.9/ (7.3–9.5)	9.3/ (8.8–10.7)	0.004*	**1.9 (1.005–2.4)**
**PDW (%)** *Median / (IQR)*	14.9/ (13.9–17.3)	15.9/ (13.5–17.7)	0.830	**0.95 (0.83–1.08)**
**PCT (%)** *Median / (IQR)*	0.22/ (0.18–0.28)	0.09/ (0.07–0.16)	< 0.001*	**0.1 (0.1–0.15)**

*PRISM* pediatric risk of mortality score, *CRP* C-reactive protein, *PLT* platelet count, *MPV* mean platelet volume, *PDW* platelet distribution width, *PCT* plateletcrit, *CI* confidence interval

Mann Whitney test for non-parametric quantitative data (expressed as median) between the two groups

^*^Significant difference at *p* value < 0.05

When we calculated platelet indices ratios, MPV/PLT, MPV/PCT, PDW/PLT, PDW/PCT, we found them to be significantly higher in the non-survivor group than the survivor group, as *p* < 0.001 in all (Table 3). Their odds ratios are presented in Table 3.

**Table 3:** Ratios of platelet indices of the children with severe sepsis according to their outcome

Variable	Survivors	Non-survivors	p	Odds ratio (95% CI)
	***n*** = 41	***n*** = 19		
**MPV/PLT** *Median / (IQR)*	0.03/ (0.019–0.08)	0.15/ (0.08–0.25)	< 0.001*	**1.01 (1.004–1.02)**
**MPV/PCT** (*Median / (IQR)*	36.25/ (29.2–49.5)	107.78/ (57.5–152.85)	< 0.001*	**3.83 (1.14–7.73)**
**PDW/PLT** *Median / (IQR)*	0.05/ (0.04–0.15)	0.23/ (0.14–0.39)	< 0.001*	**1.007 (1.001–1.01)**
**PDW/PCT** *Median / (IQR)*	67.78/ (53.1–91.11)	186.25/ (99.29–260)	< 0.001*	**2.97 (1.05–5.46)**

*PLT* platelet count, *MPV* mean platelet volume, *PDW* platelet distribution width, *PCT* plateletcrit, *CI* confidence interval

Mann Whitney test for non-parametric quantitative data (expressed as median) between the two groups

^*^Significant difference at *p* value < 0.05

When we correlated the studied markers with PRISM score, PRISM score had significant negative association with both platelet count (*r =* − 0.420, *p* = 0.001) and plateletcrit (*r =* − 0.442, *p =* 0.001). And, it had a significant positive association with CRP level (*r =* 0.497, *p =* 0.001) (Table 4).

**Table 4:** Correlations of PRISM score with CRP, platelet count and parameters

Variable	PRISM	
	r	p
**PLT**	**−0.420**	< 0.001*
**MPV**	0.047	*0.722*
**PDW**	−0.040	*0.764*
**PCT**	**− 0.442**	< 0.001*
**CRP**	**0.497**	< 0.001*

*PRISM* pediatric risk of mortality score, *CRP* C-reactive protein, *PLT* platelet count, *MPV* mean platelet volume, *PDW* platelet distribution width, *PCT* plateletcrit

Pearson’s correlation coefficient

^*^Significant level at *p* value < 0.05

Multivariate analysis was done to determine the odds ratios for platelet count, platelet indices and CRP after controlling for PRISM score, the decrease in PCT was the only risk factor reaching statistical significance, as its odds ratio was 0.101 (95% CI: 0.1–0.15) with *p* = 0.02 (Table 5).

**Table 5:** Multivariate analysis of platelet count, platelet indices and CRP after controlling for PRISM score

Variable	Odds ratio	95% CI for OR		p
		Lower	Upper	
**PLT**	0.995	0.98	1.004	0.08
**MPV**	1.405	0.80	2.4	0.2
**PDW**	0.631	0.39	1.01	0.6
**PCT**	0.101	0.1	0.15	0.02*
**CRP**	1.012	0.99	1.02	0.17

*PLT* platelet count, *MPV* mean platelet volume, *PDW* platelet distribution width, *PCT* plateletcrit, *CRP* C-reactive protein, *CI* confidence interval, *OR* odds ratio

^*^Significant level at *p* value < 0.05

**Fig. 2:**
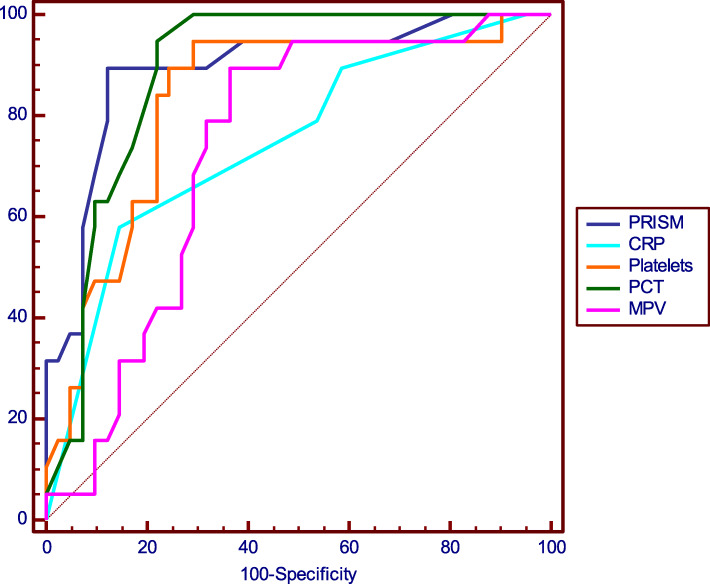
ROC curve analysis of PRISM score, CRP, platelet count and indices for prediction of death in patients with severe sepsis. PRISM: pediatric risk of mortality score, CRP: C-reactive protein, PLT: platelet count, MPV: mean platelet volume, PCT: plateletcrit

**Fig. 3:**
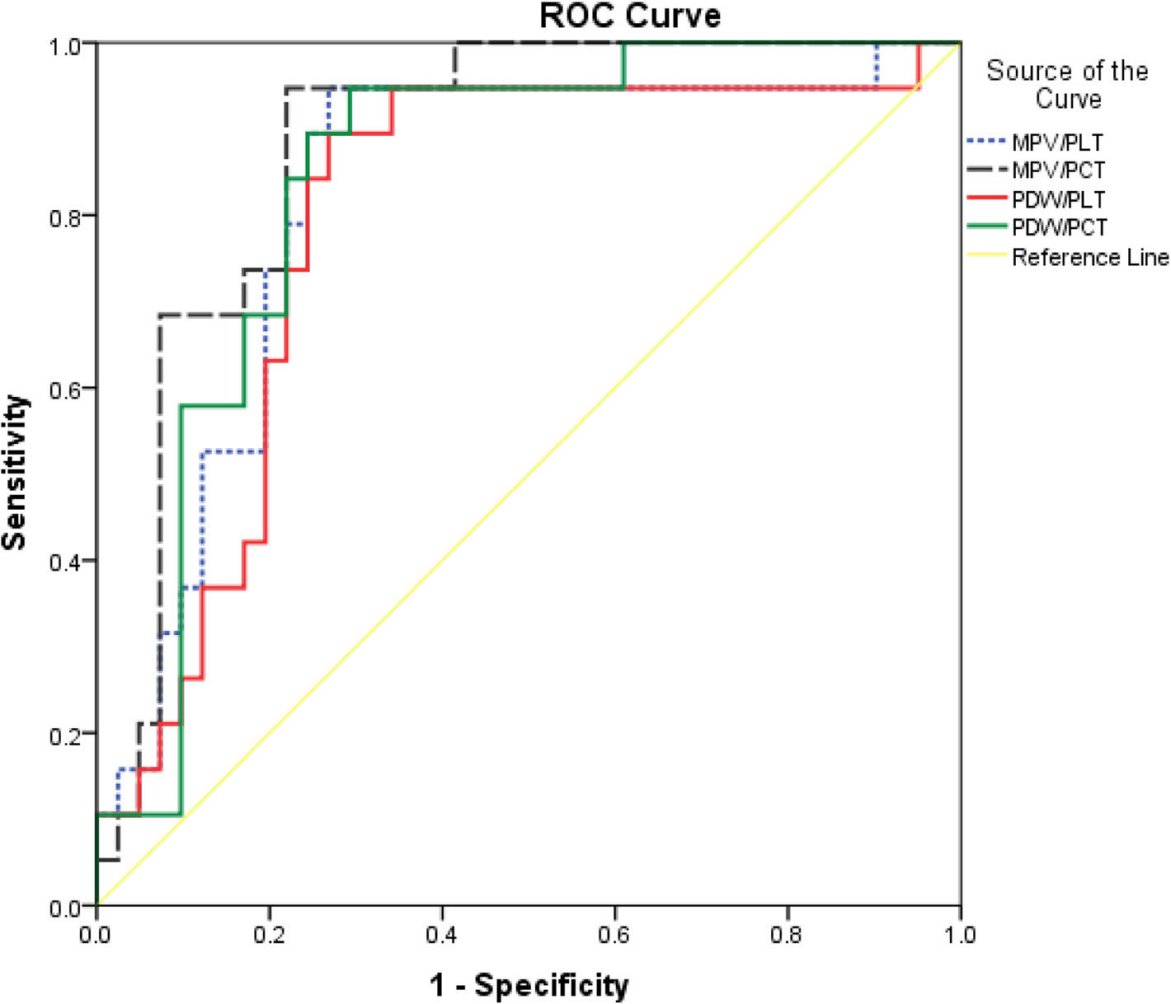
ROC curve analysis of ratios of platelet indices for prediction of death in patients with severe sepsis

ROC curve analysis of PRISM score, CRP, platelet count and indices for prediction of death in patients with severe sepsis, showed that PCT was the platelet parameter showing the largest area under the curve (AUC of 0.888), with a sensitivity of 94.74% and a specificity of 78.05% at a cut-off of ≤0.17%. The decrease in platelet count had the same sensitivity as PRISM score (sensitivity = 89.47%), taking the second place after plateletcrit regarding sensitivity of predicting death. While MPV, at cutoff point ≥8.7 fl, was the second most specific marker after PRISM score (specificity = 86.29%). MPV in this study was the least sensitive platelet parameter (sensitivity = 78.95%), but still more sensitive than CRP (sensitivity = 57.89%) (Table 6, Fig. 2). While ROC curve analysis of platelet indices ratios revealed that MPV/PCT had the largest area under the curve (AUC of 0.882), with a sensitivity of 94.7% and a specificity of 78% at a cutoff value of ≥49.8 (Table 7, Fig. 3).

**Table 6:** Predictive values of PRISM score, CRP, platelet count and indices for mortality

Variable	Optimal cutoff	AUC	Sensitivity (%)	Specificity (%)	Accuracy (%)
**PRISM**	≥ 20	0.892	89.47	87.8	88.3
**CRP (mg/dl)**	≥ 96	0.701	57.89	85.37	70.2
**Platelets (10**^**3**^**/ml)**	≤ 117	0.831	89.47	75.61	80
**MPV (fl)**	≥ 8.7	0.731	78.95	86.29	71.7
**PCT (%)**	≤ 0.17	0.888	94.74	78.05	83.3

*PRISM* pediatric risk of mortality score, *CRP* C-reactive protein, *PLT* platelet count, *MPV* mean platelet volume, *PCT* plateletcrit, *AUC* Area under curve

**Table 7:** Predictive values of ratios of platelet indices for mortality

Variable	Optimal cutoff	AUC	Sensitivity (%)	Specificity (%)	Accuracy (%)
**MPV/PLT**	≥0.076	0.823	89.5%	73.2%	78.3%
**MPV/PCT**	≥49.8	0.882	94.7%	78%	83.3%
**PDW/PLT**	≥0.14	0.795	84.2%	73.2%	76.7%
**PDW/PCT**	≥91.7	0.841	84.2%	75.6%	78.3%

*PLT* platelet count, *MPV* mean platelet volume, *PCT* plateletcrit, *PDW* platelet distribution width, *AUC* Area under curve

## Discussion

In this study, on comparing children with severe sepsis according to their outcome, the non-survivors had higher PRISM score than patients who survived. This was in accordance with many studies who found PRISM score to be higher in non survivors [17–19]. ***Pollack*** **et al.*,*** in ***2015*** stated that the increase in PRISM score is significantly associated with increase in morbidity and mortality and could estimate morbidity and mortality risk [16]. Non-survivors had also higher CRP levels than survivors. It is well known that CRP is an acute phase reactant synthesized in liver in response to infection or inflammation and its serum concentration can increase up to 1000-fold during acute inflammatory events and correlated well with severity of infection [20]. Moreover, many studies observed that CRP concentrations at ICU admission were associated with organ dysfunction, increased ICU length of stay, and higher mortality [21].

Regarding platelet count and indices, platelet count and PCT were significantly lower in non-survivor than survivors. This was in accordance with ***Venkata*** **et al.*,*** [22], who found that thrombocytopenia carries an independent risk for mortality in ICU patients and is a negative prognostic indicator for adverse clinical outcomes in ICU patients [22]. Also, a recent study stated that thrombocytopenic children at the time of admission have more likelihood of mortality than non-thrombocytopenic children in intensive care units [23]. ***Gao*** **et al.*,*** [24] found that PCT was correlated to platelet count with similar clinical implication, and they found markedly decreased PCT in patients who expired. The low platelet count in non-survivors may be attributed to the depletion of coagulation factors and platelet consumption during the septic process and the low PCT in the non-survivors may be imputed to that PCT is influenced by number and size of platelets and has a positive relationship with platelet count [24].

Meanwhile, the non-survivors had significantly larger MPV than the survivors. This situation may be caused by production of many cytokines, endothelial damage, and bone marrow suppression in septic patients [25]. A study by ***Margetic,*** in ***2012*** showed that MPV acts as an acute phase reactant in different inflammatory conditions, they stated that high MPV levels were associated with high-grade inflammation owing to the presence of large platelets in circulation [6]***.*** Two prospective studies demonstrated significant correlations between increased MPV and short-term mortality [26, 27]. Also, a study by ***Tajarernmuang*** **et al.** in ***2016,*** on adults, revealed that the gradual increase in MPV after a few days of admission was associated with increased hospital mortality [28]. An elevation of MPV suggests that the infection is invasive and uncontrolled and is related to the severity of the disease, a finding which was verified in our study, and may be useful as an assessment tool for outcome prognosis.

Furthermore, a study by ***Sezgi*** **et al.*,*** in ***2015*** showed that in patients with sepsis the MPV level was increasing during the course of the disease in non-survivors, while it was found to be decreasing in the surviving group [29].

In our study PDW increased in non-survivors than survivors, but this increase did not reach statistical significance. The PDW is increased when there is an increase in number and size of platelet pseudopodia [30]. Platelet activation causes morphologic changes of platelets, including both spherical shape and pseudopodia formation. Platelets with increased number and size of pseudopodia differ in size, which affects PDW. In a previous study, PDW was significantly higher in patients with asserted platelet activation compared with healthy persons [31].

Platelet ratios calculated in this study, MPV/PLT, MPV/PCT, PDW/PLT, PDW/PCT, were significantly higher in non-survivors than survivors. This is in accordance with a previous study in 2016 by Golwala et al., who found these ratios to be predictors of mortality in children [13].

PRISM score is a well-established score for prediction of mortality in pediatric patients. When we correlated the studied markers with PRISM score, PRISM score had significant negative associations with platelet count and plateletcrit, and it had a significant positive association with CRP level.

The negative associations of PRISM score with both platelet count and plateletcrit, confirm their negative prognostic values. Many studies addressed the relation of PRISM score and thrombocytopenia with the severity of sepsis. ***Gerardin*** **et al.*,*** in ***2018*** found that thrombocytopenic patients had higher PRISM score at admission [32]. ***Yilmaz*** **et al.*,*** in ***2013*** recommended that sequential platelet counting to identify risk as PRISM score [33]**.**

Plateletcrit was the most sensitive parameter for predicting death, with thrombocytopenia taking second place by having the same sensitivity as the PRISM score. While MPV in this study was the least sensitive platelet parameter, but it was still more sensitive than CRP, which is the most commonly used inflammatory marker to be assessed in children with sepsis. Also, MPV/PCT ratio was the most sensitive ratio to predict mortality in this study.

### Limitations

Our study has several limitations, for example, studies with larger sample size are needed. Moreover, further studies should address the changes occurring in platelet count and parameters during the course of pediatric sepsis by serial measurements of their levels during the course of the disease. Also, their association with long-term morbidity outcomes in children surviving severe sepsis syndrome should be elucidated.

## Conclusion

Thrombocytopenia is an ominous sign that should be taken seriously in pediatric sepsis syndrome. Platelet indices and their ratios are readily available, sensitive, prognostic markers, that can identify the severe sepsis patients with poorest outcome. So, we recommend platelet count, platelet indices and their ratios, especially plateletcrit and MPV/PCT ratio, should be assessed in all sepsis patients upon admission to the PICU to guide the clinical decision along with the PRISM score and CRP.

## Data Availability

The datasets analyzed during the current study available from the corresponding author on reasonable request.

## Abbreviations

MPV: Mean platelet volume
PDW: Platelet distribution width
PCT: Plateletcrit
CRP: C-reactive protein
PICU: Pediatric intensive care unit
PRISM score: Pediatric risk of mortality score
